# A Health System’s Journey toward Better Population Health through Empanelment and Panel Management

**DOI:** 10.3390/healthcare6020066

**Published:** 2018-06-15

**Authors:** Peter McGough, Vandna Chaudhari, Suzanne El-Attar, Pamela Yung

**Affiliations:** Population Health, University of Washington Medicine, Seattle, WA 98195, USA; vandna@uw.edu (V.C.); selattar@uwpn.org (S.E.-A.); pyung@uwpn.org (P.Y.)

**Keywords:** population health, empanelment, panel management, UW Medicine, value-based care, primary care

## Abstract

The USA is steadily moving towards a health system that emphasizes ‘wellness’ over ‘sickness’ care. An effective wellness program utilizes a ‘population health’ approach that ensures that all patients who seek care from a health system receive the services recommended by evidence and best practice. This means attending not just to patients who are seen for care, but also to patients who have not yet been seen. A key strategy for population health is empanelment and panel management for patients in primary care. This article relates the experience of UW (University of Washington) Medicine in implementing such a program.

## 1. Introduction 

As the USA moves towards a ‘value-based’ health care system, there is a growing emphasis on wellness and prevention programs with the intention of both improving health status and controlling associated costs. This is largely being driven by a transformation in how health care is reimbursed [1]. Until recently, physicians and health systems were ‘paid for volume’—that is, for how much health care was provided to a patient regardless of patient outcomes. At times, hospitals have been paid more for managing the failures of care, such as hospital associated infections and other complications [2].

In a value-based health reimbursement system, providers are rewarded for high quality care and potentially penalized for poor care outcomes. Initial quality care efforts were ‘visit-based’ (focused on the patients who had recently been seen by providers). This has now matured into ‘panel-based’ efforts (managing the health of all the patients assigned to a health system, regardless of visit status and whether these patients are up-to-date on recommended health measures).

These are big changes. In the previous world of volume-based care, the provision of evidence-based services, especially for screening and prevention, was haphazard at best [3]. It was often driven by the patient asking for specific services, sometimes at the encouragement of family or advertisements. When the focus shifted to visit-based care, providers began assessing the patients seen for care gaps (unmet evidence-based quality care measures). Clinic protocols were developed to forecast these care gaps and verify them with patients at their visit. Staff and physicians would address the verified care gaps with the patient at the visit or arrange for appropriate follow-up care.

What became clear in a visit-based system is that a large number of patients’ health needs were not being met—specifically those patients who had not had a recent visit. Thus, efforts were made to move from a visit-based clinical quality system to a panel-based approach. This involves monitoring the care needs of all patients assigned to a health system regardless of whether they have had a recent visit. It also requires a new perspective and a new set of tools and resources for care gap management. To move in this direction, there is support for ‘empanelment’ and ‘panel management’. Empanelment is clearly identifying all the patients assigned to a primary care provider and their clinic. Panel management is identifying the care gaps for each ‘empaneled’ patient and creating plans to address those gaps.

This paper presents the experience of an academic health system, UW (University of Washington) Medicine in Seattle, WA, USA in developing a successful population health program based on empanelment and panel management across its several primary care entities.

## 2. Materials and Methods

This case study outlines the planning and implementation of a population health strategy at a large academic health system based in Seattle, Washington—UW Medicine. UW Medicine utilized a large award from the Centers for Medicaid and Medicare Innovation (CMMI) to catalyze the transformation of primary care. As part of its population health strategy, a system-wide initiative began with the first step of empanelment, which linked primary care providers (PCPs) to the patient lives they were to be accountable for. The electronic medical record (EMR) used in the ambulatory entities of UW Medicine is EPIC, which is copyrighted and maintains control over the details of its functionalities and reporting capabilities. Each of the authors played a leadership role in one or more aspects of this initiative.

### 2.1. Empanelment—The Foundation for Population Health in Primary Care

**Figure 1:**
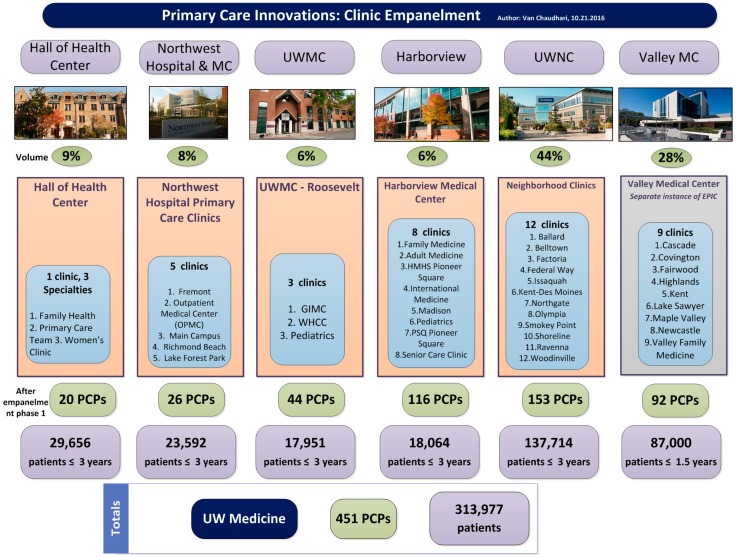
An overview of empanelment at University of Washington Medicine outlining the number of primary care providers (PCPs) at each entity, including UW Neighborhood Clinics (UWNC) & UW Medical Center ambulatory clinics.

In March 2016, UW Medicine used a large portion of a $30 million award from the Centers for Medicaid and Medicare Innovation (CMMI) to catalyze the transformation of primary care. To achieve the goals of population health, a system-wide initiative began with the foundational step of empanelment. Over the summer of 2016, UW Medicine empaneled over 450 primary care providers (PCPs) across 38 primary care clinics addressing over 310,000 patient lives (Figure 1). Empanelment identifies every primary care provider’s patient population for which they are accountable for improved health outcomes. A panel of patients includes patients who have been recently seen by their PCP or primary care clinic location, but also includes patients that have not engaged recently with their care team. For the purposes of empanelment, ‘eligible PCPs’ included attending physicians, fellows, residents, nurse-practitioners, and physician-assistants with a specialty in family medicine, internal medicine, or pediatrics. All PCPs were validated with the Clinic Chief (clinic provider leader) before the assignment of panels began. Using a variation of Murray’s four-cut empanelment algorithm and taking into account PCP, patient, and family preference, most patients with at least one or more primary care encounters with a PCP or primary care location, over a rolling three-year period, became empaneled. The exercise of empanelment was completed over four months and ultimately synchronized into the EPIC electronic medical record (EMR). 

With empanelment, each PCP was able to view key data about their panel in the EMR and assess the current state of their panel quality measures. This can help identify where opportunities exist for addressing important patient care gaps. Today, when any PCP logs into EPIC, the opening splash-page in the EMR is a display of various panel data and population health metrics. 

### 2.2. Panel Maintenance

After the initial empanelment efforts, the focus shifted to ensuring sustainable panel accuracy and avoiding degradation over time. A provider-led panel maintenance workgroup took charge of developing policies, procedures, and workflows to maintain accurate panels. The workgroup developed recommendations and sought consensus among primary care leadership to begin implementation of these procedures. One recommendation addressed when to remove a patient from a panel. It was agreed that patients without an upcoming appointment who had not seen their PCP or had a visit at their PCP’s primary care location in the past three years could be removed from the PCP’s panel (un-empaneled). Efforts would be made prior to the three-year mark to contact these patients by secure web portal message or letter at quarterly intervals to encourage them to make an appointment with their PCP. If a patient became un-empaneled, they had the option to re-establish with a PCP who has an open panel at any time.

A key recommendation was maintaining the accuracy of the General PCP field in the EMR by ensuring only empaneled clinicians (defined as ‘eligible PCPs’ above) could be listed as a General PCP and that all other clinicians with whom the patient has an established relationship (mainly specialists) became part of the ‘care team’. Work continues to educate all providers and staff that, with few exceptions, primary care clinics should be the only ones changing, adding, or removing a provider in the General PCP field.

Another important aspect of empanelment is raising awareness of the ratio of yet-to-be-empaneled or ‘un-empaneled’ patient volumes by attribution to clinic location. If the purpose of empanelment is to give every PCP and care team their slice of the primary care population, it is important to empanel as many patients as possible as they visit their PCP at the point of care. The assumption behind this principle is that the health system has open primary care capacity. Adding previously un-empaneled patients to panels raises questions around panel right-sizing and whether the system has adequate strategic capacity to absorb additional primary care lives on panels. Another area of opportunity lies in freeing up valuable PCP capacity by delegating tasks and activities better suited to the practice scope of other care team members. Striking the right work-life balance for PCPs is an important aspect of UW Medicine’s aim to improve the health of the public and achieve the Quadruple Aim. In 2018, primary care is trialing the use of monthly dashboards to show the current empanelment landscape to monitor the ratio of the un-empaneled lives versus the empaneled lives. This dashboard will help support the creation of standard processes to maintain accurate panels in the EMR in synchronization with robust workflows at the point of care.

### 2.3. Panel Right-Sizing

After empaneling all primary care providers across the UW Medicine system, a committee of primary care leaders was given the task of determining how to ‘right-size’ these panels—that is, to identify how big a PCP panel could become without becoming overwhelming. The committee was asked to review the literature on panel size as well as various data elements of the newly created panels and determine an appropriate panel size with a focus on the upper and lower boundaries of a panel. This helped determine when a panel is open or closed to new patients, which in turn provided an understanding of overall capacity and access for patients.

The analysis looked at several domains that included the impact of PCP specialty, provider FTE (full-time equivalence) and productivity, patient visit and communication activity, and patient complexity. 

The data shows a wide variability in panel sizes across the UW Medicine system entities. Some of the variability could be explained by differences in population characteristics or whether the clinic was a teaching site. However, most of the variability is not explained by measurable factors. This variability makes it difficult to have a “one size fits all” approach to panel sizes. For this reason, PCP leaders ultimately came up with two recommendations—one for teaching settings (where faculty had lower clinical FTEs) and another for predominantly clinical settings. The former recommended an average panel size of approximately 1000, while the latter proposed a panel size of around 1800. 

### 2.4. Alignment to Panel Focused Quality

In 2018, efforts were focused on aligning panel quality measures with already existing internal quality dashboards based upon patient visits. They were also trying to align with the quality goals of value-based contracts that utilize HEDIS (Healthcare Effectiveness Data and Information Set [4]) clinical quality measures, which determine the overall ratings of the health plans (e.g., STAR, a five star quality rating system used by Medicare [5]) that are reported regularly. Empanelment with panel management helps us better achieve these goals and make UW Medicine more attractive to prospective health plans with whom we would like to partner. Panel management at UW Medicine is increasingly focused on achieving targets in population metrics like the common HEDIS measures, including assessing and addressing care gaps in cancer screenings, chronic disease management, immunizations, and medication adherence. Examples of metrics tracked on the quality dashboard:

- Medicare Advantage patients who had received an Annual Wellness Visit during the year.
- Diabetic patients who had good glycemic control and retinal screening.
- Patients 50 years or older who had undergone screening for colon cancer.

### 2.5. Panel Management

Once panels were established in the EMR in early 2017, PCPs were asked to become familiar with their respective panel dashboards and answer four questions:

1. Where is the information on my panel in EPIC?
2. How is my panel doing?
3. Where are the opportunities to improve the health outcomes of my panel with my care team?
4. How can I work with leadership to ensure there is dedicated time to focus on panel management?

Panel management encompasses an important new body of work for the PCP and the care team to optimize health outcomes and patient health status. This begins with defining roles, practice scope, and designing future state workflows using EMR tools to better manage care gaps. 

### 2.6. Information Technology (IT) Resources for Panel Management

In January 2017, a new EPIC EMR Provider Dashboard was released to include the Population Health IT tools. New features included desktop tools and reports to support PCPs and care teams in viewing the status of their patient panels.

**Figure 2:**
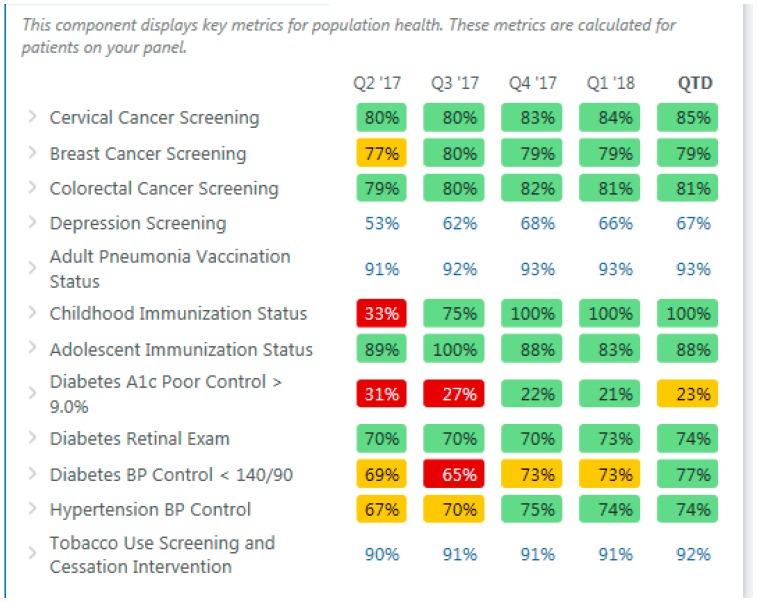
Population health metrics. BP is blood pressure.

The Population Health Metrics (Figure 2) displays information on the PCP’s entire panel and focuses on key quality metrics. PCPs and care team members can view which patients on their panel are due for screening tests, immunizations, and other important quality metrics tracked at UW Medicine along with other high priority metrics. The Population Health Metrics allows the PCP and care team to focus on individual metrics to see which patients on their panel are overdue for a preventive screening measure or not-at-goal for certain chronic disease measures such as diabetes A1C (hemoglobin A1C, a measure of glycemic control).

**Figure 3:**
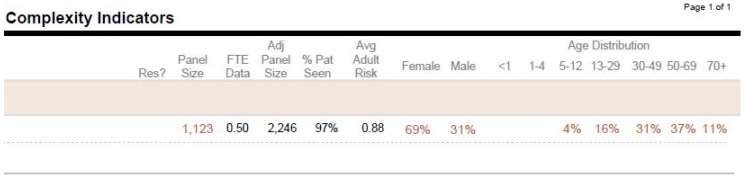
My PCP panel complexity indicators. FTE = full-time equivalence.

Another report to mention is the ‘My PCP Panel Complexity Indicators’ (Figure 3). This report provides a different high-level overview of the PCP panel. It includes total panel size, FTE-adjusted panel size (the adjusted panel size reflects the size of the panel for the PCP at 1.0 FTE), distribution of the panel demographics based on gender, age brackets, and Average Adult Risk score, which reflects panel complexity. This report could also be helpful in determining whether to keep a PCP panel open or closed to new patients.

With the implementation of these Population Health IT tools, care team workflows to optimize the use of these tools is essential for ongoing panel management. 

### 2.7. Options for Panel Management

With PCP panels in place and with desktop tools displaying the status of each provider’s panel, what happens next? One thought is that, given the desire of providers to do well, just giving the information to PCPs will drive improvement. In other words, it will happen magically.

One significant obstacle is how busy PCPs are today. Dr. Thomas Bodenheimer, a prominent U.S. physician champion for using teams in primary care, has pointed out that, “Practice improvements often fail because they rely on the willingness of physicians, who are already too busy, to take on additional work” [6]. In addition to their direct patient care activities, PCPs are also expected to effectively manage the chronic care needs of their patients along with ensuring they get the recommended preventive services. That potentially adds an additional 21 h to a 7 h day [7]. The emerging solution to this challenge is to engage the entire clinical team in support of the PCP in managing all aspects of care, including panel management.

Since panel management was a new area of work for our PCPs, we determined to do a pilot study in several of our primary care clinics where ‘team care’ was already firmly established.

### 2.8. A Team Approach to Panel Management

This team approach to the panel management pilot was performed at the UW Neighborhood Clinics, a group of twelve primary care clinics scattered in varying western Washington State neighborhoods. The majority of these clinics already utilize a team approach, which incorporates Registered Nurses, Medical Social Workers, health navigators, and others, to support the work around complex patient care management and integrated behavioral health. For this panel management pilot, seven of these clinics were selected to participate, leaving the other five clinics to serve as a control group. The key elements of the pilot included:

- Providing an hour of protected time for the PCP to meet with other clinical team members either weekly or every other week.
- The meeting was to be used for PCP panel review, and a proposed meeting agenda was shared along with draft roles and team workflows.
- PCP panel reports had been developed (see above) for the teams to utilize in their panel management activities.
- The focus of the panel management pilot included:
  (1) Coordinating Medicare Advantage Annual Wellness Visits (AWV).
  (2) Improving the management of patients with chronic conditions.
  (3) Developing strategies for addressing the screening and prevention needs of our healthier patients.

The pilot began in July 2017 and was preceded by a month of staff and provider training. Clinic check-in meetings occurred every other week to identify challenges and share potential best practices. The pilot ran for three months, with each month dedicated to one of the focus areas above. At the end of the pilot there was a formal review of the results and lessons learned, with further work planned to disseminate these panel management efforts to other clinics.

**Figure 4:**
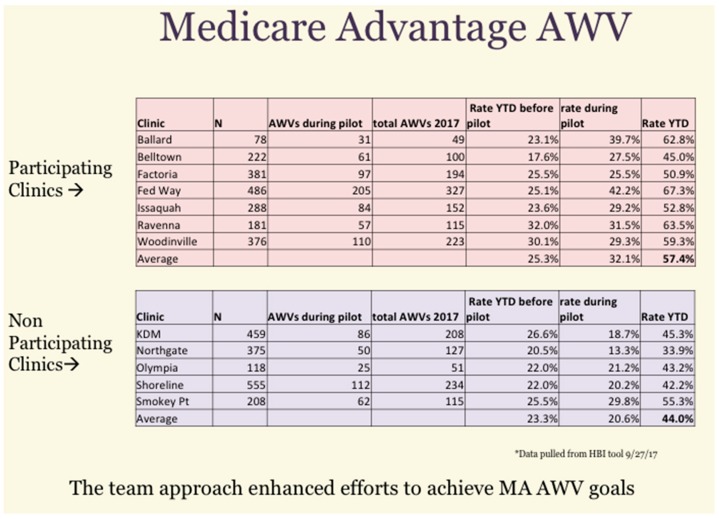
Cumulative results of a Medicare Annual Wellness Visit (AWV) initiative showing year-to-date (YTD) improvements.

Since the pilot ran for only three months it was not expected to make a major change in the overall panel quality measures. However, there was some data that suggested a significant impact. In reviewing the AWV cumulative results, the following was seen for the pilot and control clinics (Figure 4). As a group, the participating clinics achieved a higher rate of AWVs (57.4%) than did the non-participating clinics (44%) during the pilot time period.

Some of the key lessons learned from the panel management pilot:

- The pilot received very positive feedback from staff and providers—they felt it was meaningful and aligned with their priorities for patient care.
- While the pilot allotted an hour of protected time for the PCP, the impact on staff time was substantial. For every 1 h meeting it was estimated that an hour of staff time was required for meeting prep, and upwards of eight hours to manage the patient outreach tasks that came out of the meeting.
- The feedback from PCPs was that while meetings every other week were useful for the first few months, this could likely then be moved to a monthly meeting.
- In the work on improving chronic condition outcomes there were several ‘easy wins’ identified, mostly getting outside results, such as diabetic labs and eye exams. Other areas, such as improving diabetic patients with poor glycemic control, would take more time. Still other areas, such as patients with depression who had not been seen in the past year, were identified for further study.
- There was a lot of discussion on improving our outreach for screening and prevention. While some screenings (cervical cancer) required the legacy approach of scheduling patients for visits, others (breast and colon cancer screening, or encouraging patients to update their immunizations) could be accomplished without a visit. Outreach utilized both clinic and central population health staff, who worked from delegated protocols to offer selected services (e.g., stool screening for blood, scheduling a mammogram, and updating immunization status).

## 3. Discussion—The Road to Panel Management and Population Health

An African proverb advises, “If you want to go fast, go alone. If you want to go far, go together.” As UW Medicine chose to ‘go together’ towards population health, here are a few lessons learned.

Physicians and Teams: The development of effective teams in any activity requires significant cultural change, especially for physicians who are usually trained in hierarchical settings to be in charge. A strong team requires that physicians learn to respect and utilize the expertise of other members of the team, to communicate well, and work in tandem. The positive news that is often very persuasive with physicians is that working in a team can help decrease burn-out. So, while there may be some resistance initially, positive experience over time for both patients and providers in clinical teams should address this.

Get Clinical IT Help Early: Clinical IT support in solving the challenges of PCP assignment during empanelment, and later developing the desktop reports and resources to support panel management, is essential. There is often a push to do the initial planning work with clinical leadership and staff, and bring IT in later asking, “can you do this?”. By involving IT early, they can help identify potential barriers to implementation and provide important solutions to challenges that can potentially stall your empanelment initiative. 

Changing Metrics and Targets: As you move through empanelment of PCPs and begin working on the quality aspects of panel management, PCPs are often discouraged by how poor their metrics look compared with earlier visit-based reports. This shouldn’t be a surprise since this just reflects a large population of patients who have not been receiving needed care. It is important for leadership to keep the work focused, provide strategies for the different clinical areas, and provide evidence of progress.

Central versus Clinic Work: Finding the appropriate balance between the work done ‘centrally’ and that done in the clinics can be difficult. Each has an important and mutually reinforcing role. The central arm can provide needed reports and develop initiatives to identify and address care gaps. They also play a critical strategic role in identifying key system goals and priorities. On the clinic side, the work of clinical teams (‘boots on the ground’) provide essential patient outreach and support for individual patients who rely on their care team for advice and direction.

Un-empaneled Patients: Through the work of empanelment, the majority of UW patients were assigned a PCP. However, a significant minority of patients remained un-empaneled. We are still working to determine which of these patients should be empaneled and then assigning an appropriate PCP. We are also exploring if and how care gaps can be managed for un-empaneled patients. 

While it is growing clear that panel management and population health are essential to achieving the evolving goals of value-based medicine, it is also important to make the business case as the health system shifts slowly from fee-for-service to value-based models and invests in the right infrastructure to sustain primary care-based population health via panel management over time. The work described above requires capital investment during a time of financial constraints. Understanding and optimizing the opportunities in your risk-based contracts (where financial rewards are often linked to quality and population health goals) is important to health system sustainability.

Finally, population health is about improving the health status and lives of our patients. This is certainly appreciated by our patients, but is also extremely satisfying to health systems and providers. It is why we do what we do.

## 4. Conclusions

The goal of this case study was to share the current work around population health at an academic health system, and the role of empanelment and panel management in achieving the aims of population health. Significant support is required from clinical information technology in developing the decision support tools in the electronic medical record, and to develop reports of key metrics to monitor progress. A key challenge is restructuring the work required to manage patients on a PCP’s panel, harnessing skills of other clinical team members to share the work, especially the non-clinical administrative tasks associated with the work.
